# Case Report: Sudden death related to unrecognized cardiac hydatid cyst

**DOI:** 10.12688/f1000research.23277.2

**Published:** 2020-12-18

**Authors:** Med Amin Mesrati, Yosra Mahjoub, Nouha Ben Abdejlil, Marwa Boussaid, Meriem Belhaj, Hiba Limem, Ali Chadly, Abdelfeteh Zakhama, Abir Aissaoui

**Affiliations:** 1 Department of Forensic Medicine, University of Monastir, Mahdia, 5100, Tunisia; 2 Department of Pathology, University of Monastir, Monastir, 5000, Tunisia

**Keywords:** sudden death, hydatid cyst, right ventricle, autopsy, pathology

## Abstract

Echinococcosis, also known as hydatid disease, is a common parasitic human infestation found in sheep-breeding areas. It is caused by the larvae stage of *Echinococcus granulosus*, and cysts develop mostly in the lungs and the liver. Cardiac involvement is unusual and silent until acute complications or a fatal outcome occurs. Herein, we report an autopsy case of a young healthy adult who died suddenly. The autopsy revealed an external bulging on the right heart ventricle outlet with a fluid-filled cystic cavity discovered on sectioning. Dissection of other organs did not reveal other cyst locations. Histological examination ascertained the diagnosis of hydatid cyst, and death was attributed to cardiac arrhythmias. Pathologists should keep in mind that hydatid cysts can develop anywhere in the body. Solitary cardiac cyst is rare and can simulate a “silent bomb”. Unfortunately, sudden death remains the frequent manner of revelation of this disease in endemic areas.

## Introduction

Echinococcosis, also known as hydatid disease, is a parasitic human infestation that commonly occurs in countries where sheep farming is widespread, such as Mediterranean countries ^1^. In Tunisia, the prevalence and incidence of this contagion are estimated to be high (15/100000 individuals) ^2^. It is attributed to the larval stage of a tapeworm, chiefly *Echinococcus granulosus*. The mature worm inhabits the intestines of the dog and humans are accidental hosts in their life cycle. Hydatid cysts can develop anywhere in the human body, predominantly in the lung and the liver ^3, 4^. Cardiac involvement is very scarce, even in endemic regions, and its clinical evolution is asymptomatic until acute complications or a fatal outcome occurs. Herein, we report an autopsy case of a young patient who died suddenly due to an unrecognized hydatid cyst located in the right heart ventricle.

## Case report

A previously healthy 26-year-old man, without any relevant past medical family history, was discovered dead at home. A forensic medical examination and an autopsy were ordered by the judicial authorities. Information provided by the patient’s relatives revealed that the deceased was the owner of a large farm that he had been managing for the past 5 years. A few days ago, the patient had experienced mild chest pain with syncope but did not visit a cardiologist.

**Figure 1.**
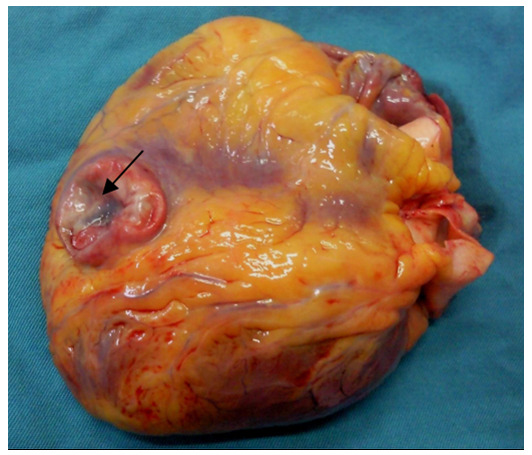
Cyst found in the right ventricle of the heart.

**Figure 2.**
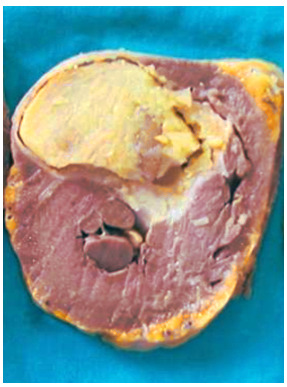
Right ventricle intra-cavitary cyst (after fixation in formalin).

During external examination, the corpse was that of a young white male, medium-build, 183cm of tall. There was no external evidence of violence or trauma, and examination of the skin revealed no rash. At autopsy, there was multi-visceral congestion without any internal haemorrhage. Internal organs were unremarkable except for the heart, which was found to be enlarged, weighing 530g (normal range: 260–350g) with an external bulging on the right ventricle outlet ( Figure 1). Sectioning of the heart revealed a fluid-filled cystic cavity, measuring 5×4cm, occupying half the volume of the right chamber and spreading to the septum. The cyst was enveloped by a thick fibrous tissue, and it featured germinative membranes, which infiltrated the myocardium. There was no hypertrophy of the myocardium. The left ventricle was 12mm of thick ( Figure 2).

**Figure 3.**
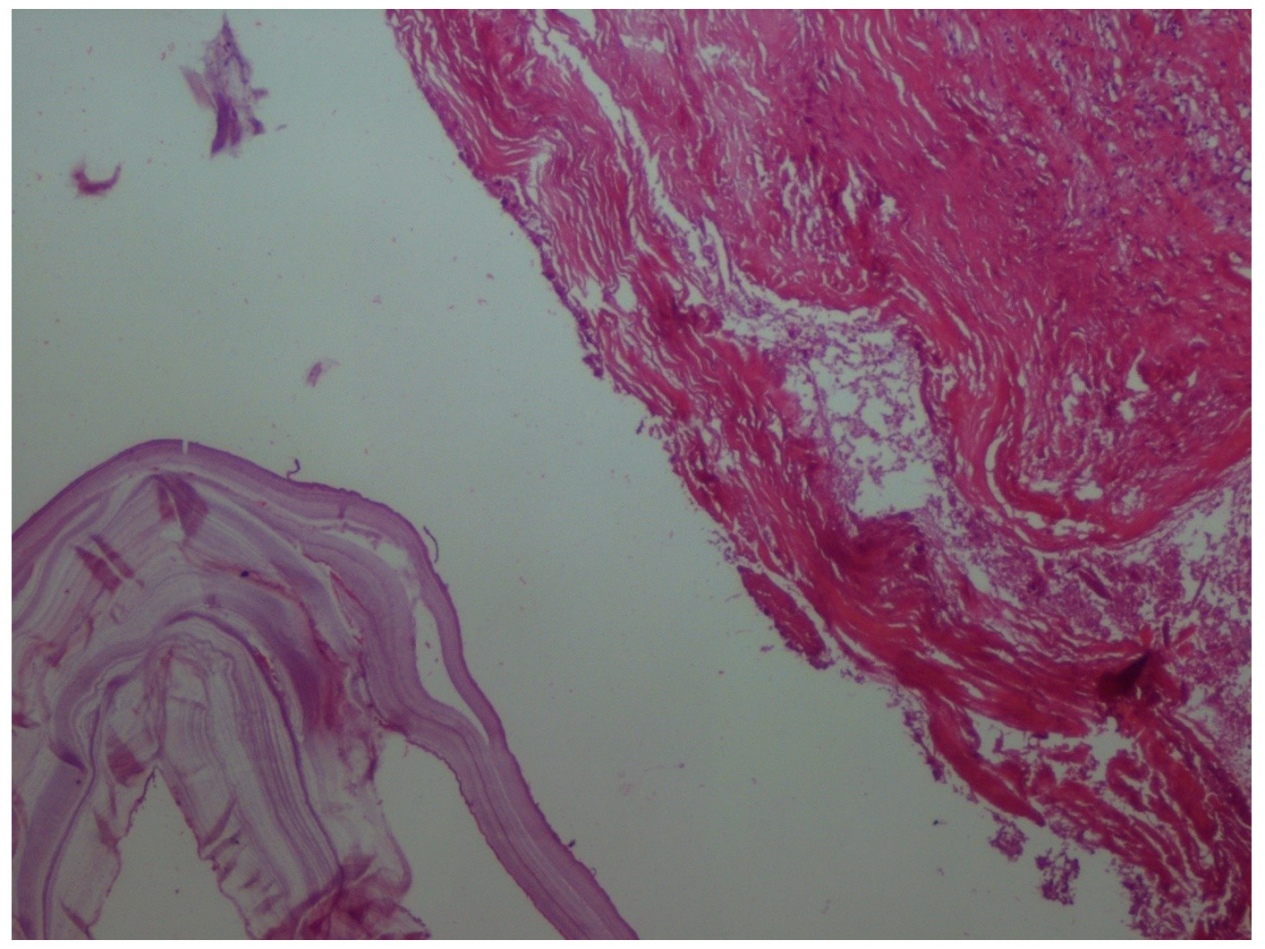
Microscopic appearance of the cyst wall. Haematoxylin and eosin, x40 magnification.

Microscopic examination using haematoxylin and eosin staining of paraffin sections of the cyst revealed classic layers of a hydatid cyst; pericyst (fibrous outer layer), ectocyst (laminated, hyaline and acellular middle layer) and endocyst (inner germinative layer) ( Figure 3). This ascertained the diagnosis of hydatid disease.

Histological findings of the left ventricle and coronary arteries were unremarkable. Examination of the lungs and liver did not reveal any abnormality, and no cysts were detected at dissection of these organs. The pulmonary arteries also did not display morphological changes, notably there were no fragments of membranes or vesicles obstructing the vessels. Toxicological screening was negative. Death was attributed to cardiac arrhythmias.

## Discussion

Hydatid disease is a parasitic infection most often induced by the larval form of the tapeworm *E. granulosus,* for which dogs are the definitive host ^5^. In rural areas, dogs are common companions for sheep farmers. Typically, dogs become infected with *E. granulosus* from eating carcasses of infected sheep in endemic areas. Adult parasites colonize the intestinal tract and the faeces of infected dogs. Humans are intermediate hosts and may accidentally acquire infection by eating contaminated food such as water or salad or after close contact, e.g. hand-to-mouth transmission, with an infected dog. After ingestion, the larvae pass through the duodenal wall, reach the portal blood system and shelter in the liver, where they are found in ~60% of cases ^6^. Some larvae may escape via the hepatic filter and cross into the pulmonary circulation, while others may continue to the systemic circulation, resulting in the generation of hydatid cysts in other organs, e.g. the lungs, muscles, bones or kidneys.

Secluded cardiac involvement by *E. granulosus* is very uncommon, and has not been described widely in the literature. Its incidence is estimated to be at about 0.4-3% of hydatid cyst cases ^7^. This scarcity is attributed to the natural resistance of heart contraction. The first described case of cardiac hydatid cyst was reported in 1836 ^8^. Most reports documented in the literature are of single cases, and a pervious literature review of the topic identified only 100 case presentations to date ^9^.

The coronary circulation is the main pathway by which the parasitic larvae reach the heart. The second route of infestation is the pulmonary vein ^10^. Due to their thickness and rich blood supply, the interventricular septum and the free wall of the left ventricular are the most common cardiac sites involved with hydatid cyst (55-60%). Pericardium, atria and right ventricle involvement have also been described; however, identification of an isolated right ventricle located hydatid cyst, like in our case, is atypical and quite rare ^11^.

Cardiac hydatid cysts are often latent. They remain symptomless and silent for a considerable time and clinical manifestation of cardiac hydatid disease may vary and depends on the size and the location of the cysts ^12^. The process of growth of cardiac cysts is slow because of the permanent traumatic action of myocardial contraction. After 2–7 years they can mimic the size of a chicken egg ^7^. Unless it’s in a critical anatomic site, cardiac hydatid disease is usually diagnosed late, as patients can manifest non-specific symptoms such as cough, palpitations and chest pain. The cyst may affect cardiac function leading to conduction and rhythmic disturbances, chest pain or angina, acute myocardial infarction or valvular dysfunction and thus pulmonary hypertension may develop ^13^. Cystic rupture is the most frequent complication (24-60%) and generally results in anaphylactic reaction with circulatory collapse. Other complications may occur leading to death, such as pericarditis, embolus, obstruction of cardiac chambers, cardiac tamponade and cardiac arrhythmias ^14^.

Hydatid cysts located in the right cardiac chambers have special features, dissimilar from those of left sided cysts. In fact, right-sided cysts, such as in our case, have a tendency to extend subendocardially and intracavitarily ^15^. In our case, the cyst seemed to extend to the septum. Cysts fissuration or rupture is more frequent, as this may trigger pulmonary embolic complication, anaphylaxis or sudden death. Chadly *et al.* ^1^ reported a case of a 22-year old man who died of pulmonary artery emboli because of the rupture of right ventricle located hydatid cyst. Pansard *et al.* ^16^ documented a case of progressive fissure of a hydatid cyst of the right ventricle, which led to a chronic pulmonary hypertension. The authors suggested that it was probably secondary to microemboli migration in the small vessels of the lung. Buris *et al.* ^17^ reported a sudden death caused by hydatid embolism in a previously healthy man who died during a race, and at autopsy hydatid cysts in the right ventricle were detected. The necropsy revealed that the cysts had embolized into the pulmonary arteries.

In our case, ventricular arrhythmia seemed to be the fatal outcome of the cardiac cyst which was found macroscopically intact. Malamou-Mitsi *et al.* ^7^ also have reported a case in which the cyst was found intact; they suggested that the death seemed to be due to fatal left ventricular arrhythmias. Singh *et al.* ^18^ reported a case of a 57-year-old man who presented with syncope due to ventricular tachycardia, and imaging revealed a right ventricular hydatid cyst. In our case, although no allergic signs were observed, anaphylactic shock cannot be excluded from the scope of mechanisms of death beyond pulmonary embolism. This fact may be explained by the rapidity and unwitnessed death.

Pathologists should keep in mind that hydatid cysts can develop anywhere in the body. Solitary cardiac cyst is rare and can simulate a “silent bomb”. Unfortunately, sudden death remains the frequent manner of revelation of this disease in endemic areas.

## Data availability

All data underlying the results are available as part of the article and no additional source data are required.

## Consent

Written informed consent for publication was obtained from the legally authorized representative of the decedent.
