# Supplementary material for: Case Report: Sudden death related to unrecognized cardiac hydatid cyst
Source: F1000Res. 2022 Sep 22;9:286. Originally published 2020 Apr 24. [Version 3] doi: 10.12688/f1000research.23277.3 (PMC7814283; doi:10.12688/f1000research.23277.3)
Supplement: Peer review history

# Peer review: Reviewer response for version 2

**DOI:** 10.5256/f1000research.31143.r76284

**Published:** 2021-01-18

**Authors:** Omer Tanyeli

**Affiliations:** 1 Department of Cardiovascular Surgery, Meram Medicine Faculty, Necmettin Erbakan University, Konya, Turkey

Thanks for the authors for the revised version of the article.

Are enough details provided of any physical examination and diagnostic tests, treatment given and outcomes?

Partly

Is the case presented with sufficient detail to be useful for other practitioners?

Partly

Is sufficient discussion included of the importance of the findings and their relevance to future understanding of disease processes, diagnosis or treatment?

Partly

Is the background of the case’s history and progression described in sufficient detail?

Yes

Reviewer Expertise:

Cardiac surgery

I confirm that I have read this submission and believe that I have an appropriate level of expertise to confirm that it is of an acceptable scientific standard.

# Peer review: Reviewer response for version 1

**DOI:** 10.5256/f1000research.25698.r69471

**Published:** 2020-08-28

**Authors:** Omer Tanyeli

**Affiliations:** 1 Department of Cardiovascular Surgery, Meram Medicine Faculty, Necmettin Erbakan University, Konya, Turkey

The cardiac hydatid disease is a relatively rare disease, especially causing symptoms in farmers. In this case report, the etiology of sudden death was attributed to possible arrhythmias caused by huge cyst in the right ventricle. 

 In the literature, the cardiac hydatid cyst is reported many times both for therapeutic options, and for its morbidity, e.g. arrhythmias. But in some Mediterranean countries, the disease is still a problem which should be kept in mind. The disease also seems to be in an increase, due to immigration/common travel policies all around the world; so that the disease can be seen in both urban and rural areas. 

 I advise the authors to also read Tanyeli *et al.* (2017 ^1^).

 Although it's not the first or the last case report in the "cardiac hydatid cyst" field, it may deserve indexing for the dramatic postmortem pictures of the heart.

Are enough details provided of any physical examination and diagnostic tests, treatment given and outcomes?

Partly

Is the case presented with sufficient detail to be useful for other practitioners?

Partly

Is sufficient discussion included of the importance of the findings and their relevance to future understanding of disease processes, diagnosis or treatment?

Partly

Is the background of the case’s history and progression described in sufficient detail?

Yes

Reviewer Expertise:

Cardiac surgery

I confirm that I have read this submission and believe that I have an appropriate level of expertise to confirm that it is of an acceptable scientific standard, however I have significant reservations, as outlined above.

## References

[1] : New World's old disease: cardiac hydatid disease and surgical principles. *Cardiovasc J Afr* .28(5) : 10.5830/CVJA-2017-006 304-308 10.5830/CVJA-2017-006 28218769PMC5756909

# Response

**Published:** 2020-08-31

**Authors:** med amin mesrati

**Affiliations:** Taher Sfar Hospital, Tunisia

Dear Tanyelli,

Thank You very much for your comments. They are so pertinent and considered by all authors.

It is true that many cases were reported in the literature about cardiac hydatid cyst but as you advanced, the exemplarity of our case remains in post-mortem pictures of the heart.

We have read your article published in this field and we considered it very interesting.

Cordially

# Response

**Published:** 2020-11-30

**Authors:** med amin mesrati

**Affiliations:** Taher Sfar Hospital, Tunisia

Dear Dr. Tanyeli,

 Thank you very much for your comments. They are so pertinent and considered by all authors.

 It is true that many cases were reported in the literature about cardiac hydatid cyst but as you advanced, the exemplarity of our case remains in post-mortem pictures of the heart.

 We have read your article published in this field and we considered it very interesting.

 It was included in our references.

 Cordially

# Peer review: Reviewer response for version 1

**DOI:** 10.5256/f1000research.25698.r63961

**Published:** 2020-06-02

**Authors:** Mejdi Ben Messaoud

**Affiliations:** 1 Cardiology A Department, Fattouma Bourguiba University Hospital, Monastir, Tunisia

*Editorial Note from F1000Research 7th September 2022 - This report has been updated to include a conflict of interest statement explaining the relationship between the reviewer and the author's institutional affiliations.*

 I read with great interest the report written by Med Amine Mesrati *et al.* about a case of sudden death due to cardiac hydatic cyst of the right ventricle. Only few similar cases were reported in the literature. The authors attributed the sudden death to ventricular arrhythmia. I think that other causes, such as anaphylactic shock (although the cyst was intact) or pulmonary embolism or right ventricular obstruction are not excluded based on the lack of some data, especially imaging exams. Additionally, other cyst locations, such as cerebral or renal, should be excluded by authors (only lungs and liver were reported) to confirm the cardiac etiology of the death.

Are enough details provided of any physical examination and diagnostic tests, treatment given and outcomes?

Yes

Is the case presented with sufficient detail to be useful for other practitioners?

Yes

Is sufficient discussion included of the importance of the findings and their relevance to future understanding of disease processes, diagnosis or treatment?

Yes

Is the background of the case’s history and progression described in sufficient detail?

Yes

Reviewer Expertise:

interventional cardiology, valvular heart disease, echocardiography, heart failure

I confirm that I have read this submission and believe that I have an appropriate level of expertise to confirm that it is of an acceptable scientific standard.

# Response

**Published:** 2020-06-02

**Authors:** med amin mesrati

**Affiliations:** Taher Sfar Hospital, Tunisia

Dear Reviewer,

Thank you for your considerations.

Response to question 1: the autopsy findings and histological tests which are always more accurate than pre-mortem imaging had excluded a pulmonary embolism or obstruction of the RV. Thus we attributed the death to cardiac arrythmias

Response to question 2: we report in the section of the case report that others organs were unremarkable. This is to say that kidney and brain didn't exhibit hydatid cyst.

Cordially
